# Raman Spectroscopy and Improved Inception Network for Determination of FHB-Infected Wheat Kernels

**DOI:** 10.3390/foods11040578

**Published:** 2022-02-17

**Authors:** Mengqing Qiu, Shouguo Zheng, Le Tang, Xujin Hu, Qingshan Xu, Ling Zheng, Shizhuang Weng

**Affiliations:** 1 Hefei Institutes of Physical Science, Chinese Academy of Sciences, Hefei 230031, China; qmq_study@126.com (M.Q.); zhengsg@hfcas.ac.cn (S.Z.); qshxu@aiofm.ac.cn (Q.X.); 2 Science Island Branch of Graduate School, University of Science and Technology of China, Hefei 230026, China; 3 Lu’an Branch, Anhui Institute of Innovation for Industrial Technology, Lu’an 237100, China; 4 National Engineering Research Center for Agro-Ecological Big Data Analysis & Application, Anhui University, Hefei 230601, China; tang_ahut@126.com (L.T.); rubber2007@126.com (X.H.); lingz0865@163.com (L.Z.)

**Keywords:** Raman spectroscopy, Fusarium head blight (FHB), wheat kernels, inception network, residual module, channel attention module

## Abstract

Detection of infected kernels is important for Fusarium head blight (FHB) prevention and product quality assurance in wheat. In this study, Raman spectroscopy (RS) and deep learning networks were used for the determination of FHB-infected wheat kernels. First, the RS spectra of healthy, mild, and severe infection kernels were measured and spectral changes and band attribution were analyzed. Then, the Inception network was improved by residual and channel attention modules to develop the recognition models of FHB infection. The Inception–attention network produced the best determination with accuracies in training set, validation set, and prediction set of 97.13%, 91.49%, and 93.62%, among all models. The average feature map of the channel clarified the important information in feature extraction, itself required to clarify the decision-making strategy. Overall, RS and the Inception–attention network provide a noninvasive, rapid, and accurate determination of FHB-infected wheat kernels and are expected to be applied to other pathogens or diseases in various crops.

## 1. Introduction

Wheat, the third largest cereal crop in terms of total production, is grown around the world and has become a staple food in Europe and Asia. Caused by *Fusarium graminearum* and *Fusarium culmorm*, Fusarium head blight (FHB) is prone to pandemics in the middle and lower Yangtze River and Jianghuai regions in China, particularly in southern Huanghuai [1]. Because FHB primarily infects the wheat ear, causing shriveled kernels with a chalky or pink color, the yield and quality of wheat are seriously threatened. The process of FHB infection is accompanied by the accumulation of toxic secondary metabolites, such as deoxynivalenol and zearalenone, which endanger human and livestock health via oxidative stress damage [2]. The detection of FHB infection of wheat kernels can ensure rational chemical control [3], guide agricultural practices, and screen FHB-resistant wheat varieties [4] and prevent and evaluate diseases as well as guarantee agricultural production safety.

Currently, visual, and biochemical methods are readily available for FHB detection [5]. The simple and intuitive visual method is performed by human experts, but its effectiveness may be reduced due to fatigue, external interference, and optical illusions [6]. Biochemical methods primarily include gas or liquid chromatography with mass spectrometry [7], polymerase chain reaction [8], and enzyme-linked immunosorbent assay [9]. Despite high specificity, they are invasive, time-consuming, and labor-intensive techniques [9,10]. These limitations force us to seek a nondestructive and rapid method with which to detect FHB-infected wheat kernels.

RS is a technique that uses inelastic scattering to obtain information about molecular vibration and rotation and provide the structure of analytes [11]. In practical applications, RS has many unique advantages compared with other rapid detection techniques, such as infrared spectroscopy and red–green–blue (RGB) imaging. First, the narrow and sharp fingerprint characteristic peaks attributed to specific or several substances have a high resolution and are typically strong [12]. In contrast, the neither marked nor clear absorbance bands of infrared spectroscopy are conducive to the analysis of changes in internal substances [13]; RGB can also only describe marked external changes in the color and texture of the tissue [14]. Then, due to the small Raman scattering cross-section of water molecules, the Raman characteristic peak of water is weak [12], which is beneficial for the analysis of biological samples [15]. In addition, portable Raman spectrometers have undergone substantial development in the past ten years, promoting the application of RS in rapid detection.

To achieve intelligent detection, RS spectra are generally combined with machine learning methods to establish determination models. Machine learning methods establish nonlinear or linear mapping between spectral and target variables and identify important factors or potential variables [12]. Partial least squares discriminant analysis (PLS-DA) [16] and orthogonal partial least squares discriminant analysis (OPLS-DA) [17] have been used for the detection of plant diseases with RS. However, these methods are relatively simple and exhibit poor fitting abilities and difficulty in excavating deep information and complex nonlinear relationships for large-scale datasets. Over the past decade, deep learning has made breakthroughs in computer vision and natural language processing due to its powerful representation learning capacity and excellent fitting ability [18]. Data-driven deep learning was proposed to learn abstract features automatically instead of manually designed or specified feature extraction [19], and to avoid the complexity, accuracy limitations, and poor stability caused by the manual feature design. Moreover, as for deep learning, the complex nonlinear relationship in large amounts of high dimensional data can be automatically fitted without the need of prior knowledge and manual intervention. Due to the simple and extensive architecture, good generalization, and excellent performance, the convolutional neural network (CNN) has become one of the most popular deep learning networks and has begun to be applied in the RS analysis of disease and toxin residues [20]. Weng et al. used RS and CNN to detect deoxynivalenol residues in FHB-infected wheat kernels with a prediction coefficient of determination of 0.9827 [21]. RS and CNN were combined to identify all 18 *Arcobacter* species from clinical, environmental, and agri-food sources with an accuracy of 97.2% [22]. In practical applications of CNNs, increasing network depth is generally used to extract abstract and precise features for better results. However, increasing depth may cause CNNs to exhibit performance saturation [23]. An Inception network, which is a novel type of CNN architecture, can capture multiple local features and reduce the number of parameters by a large width of network branches and different small sizes of convolution kernels; thus, an Inception network can improve or maintain network performance with a low calculation amount and memory occupation [24]. However, the increase in convolutional layers and convolutional kernels may lead to the Inception network suffering from a vanishing gradient and consequently lose focus on the features. The residual module transmits shallow and deep information to avoid vanishing gradients [25], and the channel attention module can dynamically adjust the nonlinear dependence relationship of each channel and has a selective channel to enhance informative channels and suppress useless features [26]. In this study, the residual module and channel attention module were combined with an Inception network to develop recognition models of FHB infection based on RS.

**Figure 1:**
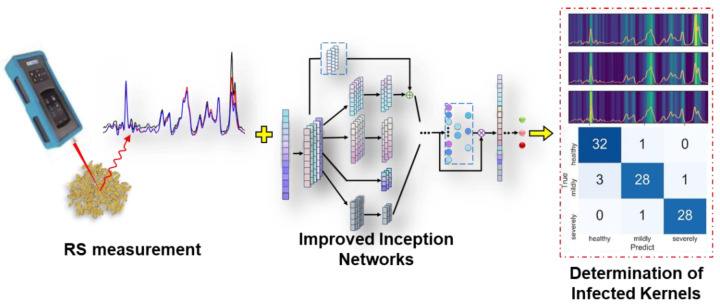
Determination of FHB−infected wheat kernels using RS combined with improved Inception networks.

This study aims to develop a determination method for FHB-infected wheat kernels using RS combined with an improved Inception network (Figure 1). The specific goals are (1) to analyze changes in RS spectra and attribution of the characteristic bands for wheat kernels infected by FHB; (2) to propose improved Inception networks with residual and channel attention modules to determine FHB infection and be compared with traditional machine learning methods; and (3) to use a feature map to visualize the feature extraction of improved Inception networks.

## 2. Materials and Methods

### 2.1. Sample Preparation

During the wheat growth period from 2018 to 2019, wheat kernel samples were collected from the Anhui Academy of Agricultural Sciences in China, where an experimental field was approximately 10 m × 10 m and divided into two equal-sized section areas. One portion of the field was inoculated with *Fusarium graminearum* and the other portion that was not inoculated served as a control group. In the early flowering period of the wheat plants, a suspension of Fusarium spores was sprayed evenly on the plants. One week after inoculation, the climatic conditions had high relative humidity and a temperature of 28–30 °C, which provided a favorable environment for the development of FHB. After reaching maturity, kernels with varying degrees of damage (healthy, mild infection, and severe infection) were harvested manually from the experimental field (Figure S1). The healthy kernels were full and had smooth and intact surfaces. Some areas of the mildly infected kernels exhibited marginal wrinkles and chalkiness. The severely infected kernels were seriously shriveled and narrow in shape and also exhibited chalkiness. In this study, a total of 467 wheat kernel samples, including 167 healthy samples, 140 mildly infected samples, and 160 severely infected samples, were examined with Raman spectroscopy.

### 2.2. Raman Spectral Measurements

After collecting wheat kernels, a handheld Raman spectrometer (B&WTEK, NanoRam^®^-1064, Newark, DE, USA) equipped with a 1064 nm laser was used to obtain Raman spectra. All spectral measurements used the following experimental parameters: an excitation wavelength of 1064 nm, an acquisition time of 60 s, a laser power of 200 mW, a spectral resolution of 4 cm^−1^, and a spectral range of 240–1736 cm^−1^. Each spectrum was the average of five scans obtained from the middle area of the wheat kernel.

### 2.3. Spectral Data Preprocessing

To minimize the impact of the experimental environment and the instrument on the Raman signal, the raw spectra data were first baseline-corrected. CH_2_ vibrations (1460 cm^−1^) could not be assigned to any specific class of compounds because this chemical group is present in many organic molecules. Raman spectra were normalized on 1460 cm^−1^ to describe the real content of biological substances.

### 2.4. Modeling Methods

#### 2.4.1. Traditional Machine Learning Methods

Support vector machine (SVM) is a small-sample machine learning method with a solid theoretical foundation [27]. SVM attempted to find an optimal decision edge that is farthest from the nearest samples of the two categories and transformed it into the solution of a convex quadratic programming problem. When the samples are linearly inseparable, SVM can project the sample onto a high-dimensional feature space through the kernel function and construct an optimal separation hyperplane. In SVM, the selection of kernel functions, kernel parameters, and penalty parameters strongly affects model performance. The parameter settings are shown in Table S1.

Random forest (RF) is a nonlinear ensemble learning algorithm that consists of multiple decision trees [28]. The training set of each decision tree is sampled by Bootstrap randomly. The input variables of each decision tree are also selected from all features. Each decision tree is regarded as a classifier, and *n* trees have *n* classification results for one sample. The RF gathers all the votes and specifies the maximum number of votes as the final output. It has advantages in handling high-dimensional data and implementing parallel processing; however, overfitting can occur easily when the dataset is full of large noise.

The gradient boosting decision tree (GBDT) is an iterative decision tree algorithm [29] that consists of several decision trees, and the predictions of all the trees are added to decide the final answer. GBDT generates a weak classifier for each iteration, each of which is trained on the residual error of the classifier of the previous round. A negative gradient of the loss function is fitted to the approximation of the residual, resulting in improved accuracy of the final classifier. GBDT can manage nonlinear information, but the interdependence between trees increases computational complexity and training time.

#### 2.4.2. Inception Networks

CNN is essentially a multilayer perceptron and can automatically learn the mapping relationship between raw data and labels of samples. With the aid of sparse connections and weight sharing, the convolution layer of the CNN can obtain the local features, which markedly reduces the number of parameters and the possibility of overfitting. In short, due to its powerful feature learning ability, easily extensible structure, and effective determination performance, CNN has widely been used in many fields, such as image classification and semantic segmentation.

**Figure 2:**
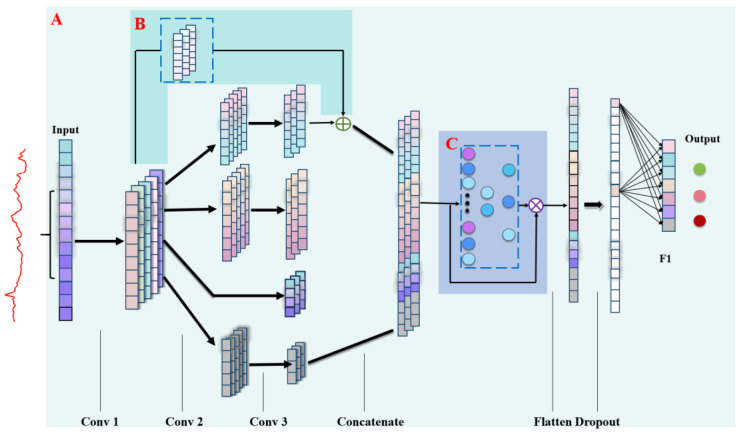
Structures of improved Inception networks: Inception (**A**); residual module (**B**); channel attention module (**C**). The blocks in the dotted boxes are added or subtracted based on the experiments.

Inception network is an effective CNN architecture that is developed by increasing both the width and depth of a neural network. Parallel convolution operations were performed on feature maps to extract different-scale information and then concatenated into deep features. The architecture can retain or improve model performance; however, the stacked layers will markedly increase the number of training parameters and required computational resources and easily cause vanishing gradients and defocused areas to develop with important features. In this study, a residual module and channel attention module were used to mitigate these problems. The residual module allows the information of the shallow layer to be transmitted directly to the deep layer. The channel attention module assigns different weights to each channel by multiplication and makes the network consider only important features. The structures of various improved Inception networks are shown in Figure 2 and the parameter settings are shown in Table S2. The network body consisted of three sets of convolution operations: a flattened layer, a dropout layer, and a dense layer for classification. The Conv 2 block of the Inception network (Figure 2A) contained two 1 × 1 convolution layers and a pooling layer, which was used to limit the number of input channels. With the convolution of different kernels (1 × 1, 1 × 3 and 1 × 5) in Conv 3, the outputs of the Conv 2 block became the feature information with different scales and were then concatenated together. The residual module (Figure 2B) connected the output of the Conv 1 convolution layer with the single-scale information in the Conv 3 block. A 1 × 1 convolution was used to ensure that the dimensions of the input and output were consistent. The channel attention module (Figure 2C) used global average pooling to generate statistics for each channel and used two fully connected layers and a Sigmoid function to learn the weights between feature channels. In addition, the focus of important features were enhanced.

### 2.5. Performance Evaluation

Healthy, mild, and severe FHB-infected wheat kernels were divided into a training set, validation set, and prediction set at a ratio of 3:1:1. TensorFlow 1.14.0 and Keras 2.2.4. were used to build the improved Inception network. Based on Scikit-learn 0.21.3, RF, SVM, and GBDT were implemented. All methods were run on a computer equipped with an Intel Core i7 and a Geforce GTX1650. The accuracy of correct classification (*ACC*) of the training set (*ACC_T_*) and validation set (*ACC_v_*) was applied to parameter adjustment and preliminary network evaluation. Finally, *precision*, *recall*, *F1-score*, and *ACC* of the prediction set (*ACC_P_*) were used to evaluate model performances.

## 3. Results and Discussion

### 3.1. Raman Spectra of Wheat Kernels

**Figure 3:**
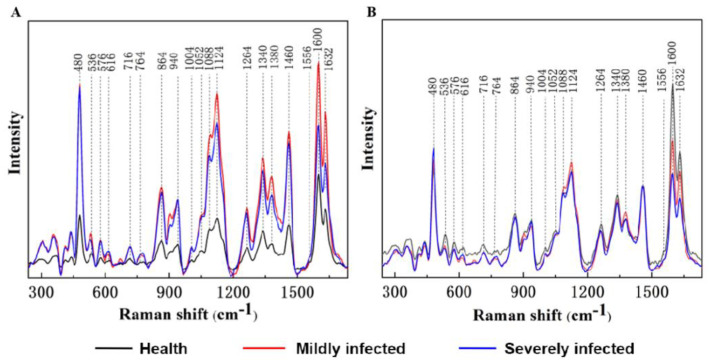
Raw Raman spectra (**A**) and Raman spectra normalized by the peak at 1460 cm^−1^ (**B**) of healthy wheat kernels, mildly FHB−infected kernels, and severely FHB−infected kernels.

Raman spectra of healthy, mildly FHB-infected, and severely FHB-infected wheat kernels were measured and are shown in Figure 3A. A series of sharp and strong peaks appeared in the vibration bands of lignin, carotenoids, pectin, cellulose, and starch (Table 1). Lignin had two vibration peaks at 1600 and 1632 cm^−1^, and the peak at 1600 cm^−1^ can be attributed to C-C ring stretching and symmetric C-H vibration, while the vibration band of 1632 cm^−1^ can be attributed to the C-C aromatic ring vibration [30]. The vibrational band of pectin compounds at 864 cm^−1^ can be attributed to the C-O-C skeletal mode of glycosyl bonds [31]. There were two vibration bands at 1095 and 1120 cm^−1^, both of which can be attributed to the C-O-C of cellulose and C-O-H vibrations of glucose [32]. The Raman peaks of the protein centered at 1632, 1556, and 1264 cm^−1^ can be attributed to the carbonyl vibration of the peptide bond [33,34]. The peaks at 480, 536, 864, 940, 1052, 1264, and 1340 cm^−1^ can be attributed to starches and monosaccharides, which were related to the vibrations of C-C-O and C-O-H [32].

Due to the marginal shift of the laser distance caused by the uneven surface of wheat, it was difficult to describe the relationship between different infections from Raman spectra after baseline correction. Considering that -CH_2_ exists and is stable in many organic molecules, its Raman peak at 1460 cm^−1^ should remain constant under a similar measurement to normalize the Raman spectra to describe the effect of FHB infection [35] (Figure 3B). As shown in the figure, some changes in the intensity and wavenumber of the vibration bands of lignin, carotenoids, pectin, cellulose, protein, and starch appeared in healthy, mildly infected, and severely infected wheat kernels. The intensity of the Raman peaks of lignin at 1600 and 1632 cm^−1^ gradually decreased, indicating the degradation of lignin produced by lignin-degrading enzymes secreted from fungi [36]. In mildly infected wheat, a marked decrease was observed in the peak at 1556 cm^−1^, which was associated with the rapid decomposition of carotenoids caused by the carotenoid lyases generated by fungi [37,38]. However, the intensity at 1556 cm^−1^ of severely infected kernels exhibited no significant changes primarily because the decomposition rate of carotenoids slowed down. The Raman peak of pectin at 864 cm^−1^ exhibited a similar trend to carotenoids. Pectinase is the primary cell wall-degrading enzyme secreted by fungi and bacterial pathogens and is regarded as an important toxic factor for fungal attack [39]. Results showed that fungal infection induced enzyme activity, leading to a reduction in pectin concentration, particularly in mild periods. Cellulose, a macromolecular polysaccharide composed of glucose, is the most abundant structural component in the primary cell wall of wheat kernels. Due to the differences in the composition and content of cell walls, the wavenumbers of cellulose at 1095 and 1120 cm^−1^ were marginally shifted to 1088 and 1124 cm^−1^ [40]. In mildly infected kernels, the intensity of bands at 1088 and 1124 cm^−1^ increased rapidly, revealing that cellulose was hydrolyzed to produce glucose molecules. The decrease in peak intensity for severely infected kernels shows that FHB promoted the conversion of glucose into polymeric hydrocarbons. Raman spectra of proteins typically exhibited several amide vibrations, known as amide I (1640–1670 cm^−1^), amide II (~1555 cm^−1^), and amide III (1230–1270 cm^−1^) [40]. In the spectra of FHB-infected wheat kernels, the intensity of 1632, 1556, and 1264 cm^−1^ markedly decreased, which demonstrated that the growth of pathogens promoted the hydrolysis of amide bonds in protein. Carbohydrates composed of monosaccharides and starches are major tissue components of wheat kernels. A slow increase of 480 cm^−1^ resulted from the combined effects of the gradual hydrolysis of starch and the accumulation of monosaccharides. For mild infection, the vibration bands of starch at 536, 864, 940, 1052, 1264, and 1340 cm^−1^ markedly decreased, suggesting that amylase activity was enhanced via the induction of fungi to accelerate the hydrolysis of starch. In contrast, the enzyme activity was weakened by severe infections. Thus, based on these changes in the Raman peaks, the feasibility of RS in the detection of FHB-infected wheat kernels was described in general.

### 3.2. Analysis of FHB Infection Using Traditional Machine Learning Methods

**Figure 4:**
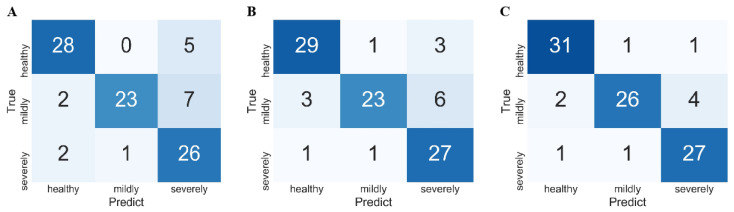
Confusion matrix of RF (**A**); GBDT (**B**); SVM (**C**).

RF, GBDT, and SVM were used to construct classification models of FHB-infected wheat kernels (Table 2), and the parameter settings are shown in Table S1. First, RF achieved the worst results of *ACC_T_* = 100%, *ACC_V_* = 82.98%, and *ACC_P_* = 81.91%, and light overfitting appeared probably because RF was sensitive to the background noise of spectra. The *recall* and *precision* of healthy kernels were 84.85% and 87.5%, respectively. Healthy kernels could be identified accurately, and other categories were less recognized as healthy kernels (Figure 4A). The *recall* of mildly infected kernels was only 71.88%, while the *precision* was as high as 95.58%. These kernels thus tended to be misclassified as healthy and severely infected kernels, while the other categories were rarely misclassified as mildly infected kernels (Figure 4A). The *recall* of severely infected kernels was as high as 89.66%, while the *precision* was only 68.42%. Most severely infected kernels were correctly classified, but many healthy and mildly infected kernels were identified as severely infected kernels (Figure 4A).

Next, GBDT achieved a relatively good classification of *ACC_T_* = 100%, *ACC_V_* = 85.11%, and *ACC_P_* = 84.04%. Compared with RF, the *precision, recall*, and *F1-score* of healthy and severely infected kernels were markedly higher, which indicated that the ability to recognize the two categories was enhanced (Figure 4B). The *precision*, *recall*, and *F1-score* of mildly infected kernels decreased marginally due to the similar feature information with severely infected kernels.

SVM performed better than GBDT, with *ACC_T_* = 96.77%, *ACC_V_* = 90.42%, and *ACC_P_* = 89.36%. The *F1-scores* of 92.54%, 86.67%, and 88.52% showed good predictive ability for the identification of healthy, mildly infected, and severely infected kernels because support vectors determined the decision boundary and avoided the interference of outliers and noise. SVMs with linear kernel functions were better than nonlinear classifiers, such as RF and GBDT. This result showed that the spectra of FHB-infected wheat kernels were easier to distinguish by linear models after mapping high-dimensional space. Traditional machine learning methods combined with RS can thus discriminate FHB-infected wheat kernels, but recognition accuracy must be improved to meet the requirements of practical applications, particularly for mild infections (Figure 4C).

### 3.3. Analysis of FHB Infection Using Inception Networks

**Figure 5:**
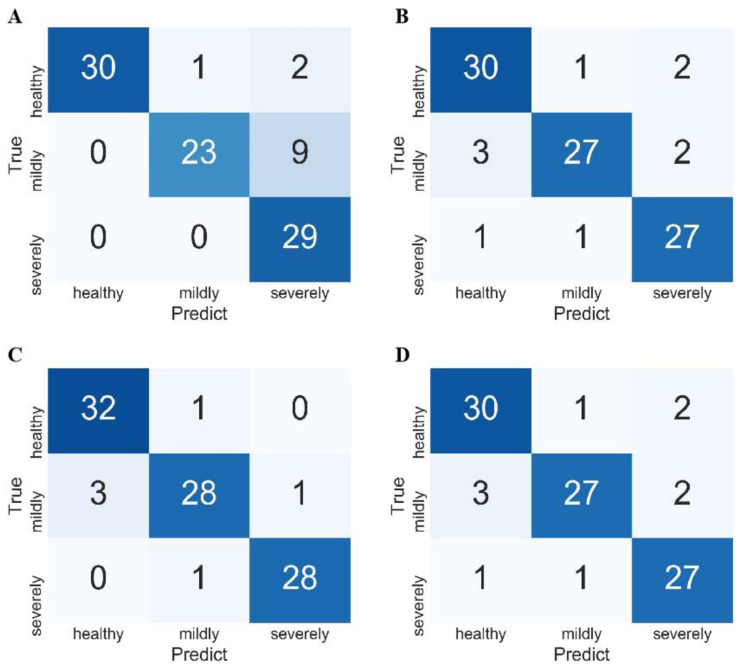
Confusion matrix of Inception (**A**); Inception–residual (**B**); Inception–attention (**C**); Inception–residual–attention (**D**).

To improve the recognition of FHB infections, deep networks were used to develop identification models for RS spectra. Specifically, based on the Inception network and combined with the residual module and channel attention module, four networks, namely Inception, Inception–residual, Inception–attention, and Inception–residual–attention were constructed with the parameter settings shown in Table S2. For the Inception network, parallel convolution kernels with different sizes were used to identify fusion information at different scales, and a small convolution kernel was used to increase computation speed and mitigate overfitting. The designs allowed the Inception network to achieve better results than RF and GBDT with *ACC_T_* = 100%, *ACC_V_* = 92.56%, and *ACC_P_* = 87.23% (Table 3). However, the Inception network poorly identified mild and severe wheat kernels, achieving a *recall* of 71.88% and a *precision* of 72.50%. These results indicated that some mildly infected kernels were not selected and were misclassified as severely infected kernels (Figure 5A). These unsatisfactory results may be attributed to the fact that the loss function was difficult to train and converge for deeper and wider networks and focus on critical features.

Subsequently, a residual module was used to build the Inception–residual network, and better results were obtained with *ACC_T_* = 100%, *ACC_V_* = 89.36%, and *ACC_P_* = 89.36%. However, the *recall* of mild infections and the *precision* of severe infections were only 84.38% and 87.10%, respectively, exceeding the Inception network, while the other evaluation indicators of the prediction set decreased marginally. Although the residual module enhanced the ability to recognize mild infections, it further suffered from a lack of focus on features for other infections (Figure 5B).

Because the attention mechanism can strengthen the focus of network features, the Inception–attention network achieved the excellent classification of *ACC_T_* = 97.13%, *ACC_V_* = 91.49%, and *ACC_P_* = 93.62%, and the *precision*, *recall*, and *F1-score* of healthy, mildly infected, and severely infected wheat kernels were better than those of the other Inception networks. In particular, the *recall* and *F1-score* of mildly infected wheat kernels were 87.50% and 90.32%, respectively, indicating that the Inception–attention network can provide more accurate recognition of mildly infected wheat kernels (Figure 5C). This result was primarily caused by the channel attention module that allowed the network to selectively enhance channels with large amounts of information and suppress unimportant channels by learning the weight of each channel.

Combined with the residual module and the attention module simultaneously, the Inception–residual–attention network was expected to perform a more accurate analysis. Unfortunately, *ACC_T_* = 99.28%, *ACC_V_* = 89.36%, and *ACC_P_* = 90.43% were marginally inferior to those of the Inception–attention network, indicating that the complex combination model may not achieve better expression (Figure 5D).

### 3.4. Feature Visualization of the Inception–Attention Network

**Figure 6:**
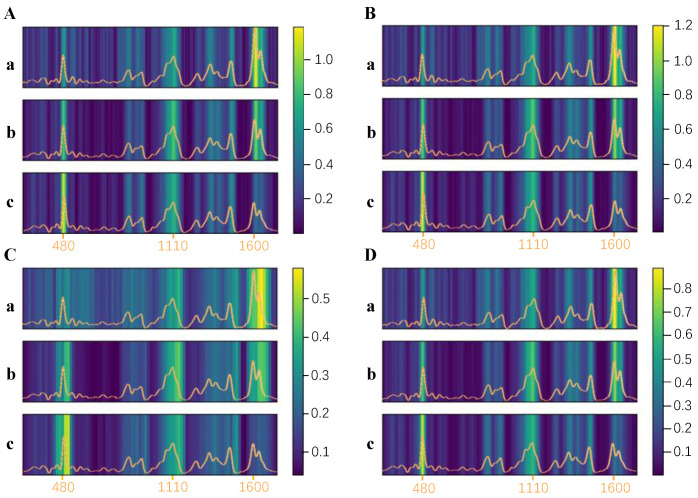
Feature maps of four parallel convolution layers (**A**–**D**), healthy wheat kernels (**a**); mildly infected wheat kernels (**b**); severely infected wheat kernels (**c**). The insets are the spectra of the corresponding types of kernels, and orange colored X-coordinates represent the Raman shift (cm^−1^).

Model interpretation was used to identify features that contributed to the results clearly and to help researchers understand the modeling process. In particular, the interpretation of black-box models such as CNNs is a challenging task. The average feature map of the channel can be used to visualize the extracted features of CNN, and the average feature map of four parallel convolution kernels in the Inception–attention network were calculated as shown in Figure 6. In the figure, the brightness indicates the criticality of the spectral region: the higher the brightness, the more critical the spectral region. The important spectral regions were distributed at 450–510 cm^−1^, 1080–1140 cm^−1^, and 1590–1650 cm^−1^, and thus made strong contributions to the model. Figure 6A–D shows that the feature maps of the four parallel convolution kernels all focus on the same spectral region, further confirming the importance of these regions. The trends of pixel brightness in important spectral regions highlighted the differences in the spectral intensities for different infection severities (all (a–c) in Figure 6). The brightness at 480 cm^−1^ indicated that the number of monosaccharides gradually increased. With the brightness increasing at first and then decreasing at 1088 and 1124 cm^−1^, cellulose was gradually hydrolyzed into glucose, and then glucose was decomposed into hydrocarbons. The decreased brightness at 1600 and 1630 cm^−^^1^ indicated that the lignin gradually degraded. Therefore, monosaccharides, cellulose, glucose, and lignin were deemed to be the most critical factors in the Inception–attention network. In the case of monosaccharides and lignin, for example, Egging’s study found that the intensity of the vibrational spectra of sugars first increased and then decreased and the intensity of the 1600 cm^−1^ band that originated from lignin decreased after the mold infection of wheat [41], which were consistent with the above results. The interpretation of the model verified the above qualitative spectral analysis. With the development of portable Raman spectrometers and embedded computing, more accurate and elaborate analysis can be achieved when studying plant diseases.

In recent years, some researchers have explored the identification of plant diseases based on RS. Sanchez et al. combined RS with OPLS-DA to detect Huanglongbing-infected citrus leaves at early and late stages [42]. The accuracies of early- and late-stage leaf infections were 83.3% and 100%, respectively. However, the OPLS-DA model could not accurately recognize early infections, requiring infection stages to be further divided. Zhao et al. determined the degrees of sclerotinia disease in intact rape leaves based on LS-SVM with PCA [43]. Due to the limited number of collected samples, LS-SVM may perform poorly in fresh samples. Mandrile et al. explored the application of RS for tomato leaves inoculated with tomato yellow leaf curl Sardinia virus (TYLCSV) and tomato spotted wilt virus (TSWV) at different time periods [44]. The PLS-DA classification model achieved a sensitivity, specificity, and accuracy that were greater than 75%. Compared with these studies, the Raman technique was used to determinate FHB-infected wheat kernels for the first time in this study, and its applicability was preliminarily demonstrated by analyzing the spectral changes and band attribution of lignin, carotenoids, pectin, cellulose, protein, and starch in healthy, mildly infected, and severely infected wheat kernels. Moreover, a powerful analysis model with high accuracy and good generalizability was obtained by the Inception–attention network. However, the recognition accuracy remains insufficient in actual scenarios to describe subtle and complex associated spectral differences due to the general sensitivity of RS and the lack of key indicators. Enhancing the RS signal, adopting advanced and novel Raman techniques, and screening direct and high-correlated analysis indicators may be feasible and effective approaches to improve the determination of FHB-infected wheat kernels.

## 4. Conclusions

RS and improved Inception networks were used to determinate FHB-infected wheat kernels. For wheat kernels with different infection severities, changes in Raman bands that were attributed to the internal components of lignin, carotenoids, pectin, cellulose, and starch were easily observed. By combining the residual module and attention module, improved Inception networks were constructed to develop determination models. The Inception–attention network achieved the best prediction of *ACC_T_* = 97.13%, *ACC_V_* = 91.49% and *ACC_P_* = 93.62% among all the models. Finally, the average feature map of the channel visualized important information in feature extraction and explained the decision-making strategy of the Inception–attention network. Therefore, the combination of RS and the Inception–attention network can accurately and rapidly identify FHB-infected wheat kernels and provide useful guidance for the identification, assessment, and prevention of other crop diseases. However, for practical applications, the identification of wheat kernels must be improved due to the inconspicuous spectral differences and a lack of key indicators induced by the complex composition of wheat kernels. In the future, we believe that the innovation of RS technology, accumulation of samples, refinement of analysis, and development of modeling methods will be used to help mitigate these limitations.

## Figures and Tables

**Table 1:** Vibrational bands and their assignments in Raman spectra of wheat kernels.

Band	Vibrational Mode	Assignment
480	C-C-O and C-C-C deformations; related to glycosidic ring skeletal deformations	Carbohydrates
	δ(C-C-C) + τ(C-O) scissoring of C-C-C and out-of-plane bending of C-O	
536	S-S gauche-gauche-trans	Protein
576	δ(C−C−O) + τ(C−O)	Carbohydrates
616	δ(C-C-O) of carbohydrate	Carbohydrates
716	δ(C-C-O) related to glycosidic ring skeletal deformations	Carbohydrates
764	δ(C-C-O)	Carbohydrates
864	δ(C-C-H) + δ(C-O-C) glycosidic bond; anomeric region	Carbohydrates
	(C-O-C) skeletal mode of α-anomers	Pectin
940	Skeletal modes; δ(C-O-C) + δ(C-O-H) + ν(C-O)α-1,4 glycosidic linkages	Carbohydrates
1004	ν_3_(C-CH3 stretching) and	Carotenoids
	phenylalanine	Proteins
1088	ν(C−O) + ν(C−C) + δ(C−O−H)	Carbohydrates
1124	ν(C−O) + ν(C−C) + δ(C−O−H)	Carbohydrates
1264	ν(C−O) + ν(C−C) + δ(C−O−H)	Carbohydrates
	Guaiacyl ring breathing, C-O stretching (aromatic)	Lignin
1342	ν(C−O); δ(C−O−H)	Carbohydrates
1380	δ(C−O−H), coupling of the CCH and	Carbohydrates
	COH deformation modes	
1460	δ(CH) + δ(CH2) + δ(C−O−H) CH, CH2,	Carbohydrates
	and COH deformations	aliphatic
		Lignin
1556	–C=C– (in plane)	Carotenoids
1600	ν(C–C) aromatic ring + σ(CH)	Lignin
1632	C=C–C (ring) or C=O stretching, amide I	Lignin
		Proteins

**Table 2:** Classification of FHB-infected wheat kernels using RF, GBDT, and SVM.

Methods	Classes	Accuracy (%)	Prediction Set		
			*Precision* (%)	*Recall* (%)	*F1-Score* (%)
RF	Healthy	*ACC_T_* = 100	87.50	84.85	86.15
	Mildly infected	*ACC_V_* = 82.98	95.58	71.88	82.14
	Severely infected	*ACC_P_* = 81.91	68.42	89.66	77.61
GBDT	Healthy	*ACC_T_* = 100	87.88	92	87.88
	Mildly infected	*ACC_V_* = 85.11	87.88	71.86	80.70
	Severely infected	*ACC_P_* = 84.04	87.88	93.10	83.08
SVM	Healthy	*ACC_T_* = 96.77	91.18	93.94	92.54
	Mildly infected	*ACC_V_* = 90.42	92.86	81.25	86.67
	Severely infected	*ACC_P_* = 89.36	84.38	93.10	88.52

Abbreviations: RF, random forest; GBDT, gradient boosting decision tree; SVM, support vector machine; *ACC*, accuracy of correct classification; *ACC_T_*, *ACC* of the training set; *ACC_V_*, *ACC* of the validation set; *ACC_P_*, *ACC* of the prediction set.

**Table 3:** Classification of FHB-infected wheat kernels using Inception, Inception–residual, Inception–attention and Inception–residual–attention networks.

Networks	Classes	Accuracy (%)	Prediction Set		
			*Precision* (%)	*Recall* (%)	*F1-Score* (%)
Inception	Healthy	*ACC_T_* = 100	100	90.91	95.24
	Mildly infected	*ACC_V_* = 92.56	95.83	71.88	82.14
	Severely infected	*ACC_P_* = 87.23	72.50	100	84.06
Inception–residual	Healthy	*ACC_T_* = 100	88.24	90.91	89.56
	Mildly infected	*ACC_V_* = 89.36	93.10	84.38	88.52
	Severely infected	*ACC_P_* = 89.36	87.10	93.10	90
Inception–attention	Healthy	*ACC_T_* = 97.13	91.43	96.97	94.12
	Mildly infected	*ACC_V_* = 91.49	93.33	87.50	90.32
	Severely infected	*ACC_P_* = 93.62	96.55	96.55	96.55
Inception–residual–attention	Healthy	*ACC_T_* = 99.28	88.57	93.94	91.18
	Mildly infected	*ACC_V_* = 89.36	90	84.38	87.10
	Severely infected	*ACC_P_* = 90.43	93.10	93.10	93.10

Abbreviations: *ACC*, accuracy of correct classification; *ACC_T_*, *ACC* of the training set; *ACC_V_*, *ACC* of the validation set; *ACC_P_*, *ACC* of the prediction set.

## Data Availability

All data are contained within the article.

## Supplementary Materials

The following are available online at https://www.mdpi.com/article/10.3390/foods11040578/s1, Figure S1: Images of wheat kernels with varying degree of damage, Table S1: Parameter setting of different classification models, Table S2: Parameter setting of different networks.
