# Supplementary material for: Raman Spectroscopy and Improved Inception Network for Determination of FHB-Infected Wheat Kernels
Source: Foods. 2022 Feb 17;11(4):578. doi: 10.3390/foods11040578 (PMC8870785; doi:10.3390/foods11040578)
Supplement: Supplementary file 1 [file foods-11-00578-s001.zip › foods-1568230-supplementary.pdf]

Raman Spectroscopy and Improved Inception Network for Determination of FHB-Infected Wheat Kernels

Mengqing Qiu [1,2], Shouguo Zheng [1,3], Le Tang [4], Xujin Hu [4], Qingshan Xu [1], Ling Zheng [4], Shizhuang Weng [3,4,*]

[1] Hefei Institutes of Physical Science, Chinese Academy of Sciences, Hefei 230031, China
[2] Science Island Branch of Graduate School, University of Science and Technology of China, Hefei 230026, China
[3] Lu'an Branch, Anhui Institute of Innovation for Industrial Technology, Lu'an 237100, China
[4] National Engineering Research Center for Agro-Ecological Big Data Analysis & Application, Anhui University, Hefei 230601, China
* Correspondence: weng_1989@126.com; Tel.: +86-13695601875

Supporting Information

Three kinds of wheat kernels

Wheat kernels of different FHB-infected degree (healthy, mild infection and severe infection) were obtained (**Figure S1**) from the experimental field of Anhui Academy of Agricultural Sciences in Anhui province, China.

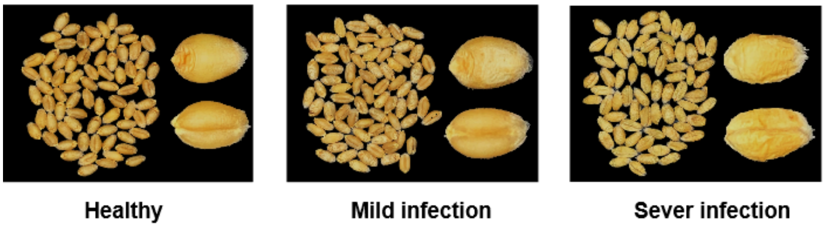

Figure S1. Images of wheat kernels with varying degree of damage.

Parameter setting of models

The parameter settings for the RF, GBDT, SVM models were shown in **Table S1**.

Table S1. Parameter setting of different classification models.

Methods	Parameters
**RF**	'n_estimators': 100,'max_depth=18', 'max_features': 'auto', 'max_depth': None
**GBDT**	'learning_rate=1.1', ''n_estimators=100', 'criterion = 'friedman_m''

**SVM**	'C = 0.5', 'kernel = 'linear'','probability = True'

RF:

n_estimators — Number of decision trees in a random forest.

max_depth — Maximum depth of the decision tree.

max_features — Maximum feature number of random forest partition.

GBDT:

n_estimators — The number of boosting stages to perform.

criterion — The function to measure the quality of a split.

SVM

C— Regularization parameter.

kernel — Specifies the kernel type to be used in the algorithm.

probability — Whether to enable probability estimates.

Parameter setting of networks

The parameter settings for the Inception, Inception-residual, Inception-attention and Inception-residual-attention networks were shown in **Table S2.**

Table S2. Parameter setting of different networks.

Methods	Parameters
**Inception**	Conv1(Relu)8@7×1<br>Conv2_1(Relu)8@1×1 Conv2_2(Relu)8@1×1 Max-pooling 3×1<br>Conv3_1(Relu)5@1×1 Conv3_2(Relu)5@1×1<br>Conv3_3(Relu)5@3×1 Conv3_4(Relu)5@5×1<br>Concatenate(Conv3_1 ,Conv3_2, Conv3_3, Conv3_4)<br>Flatten 3235 Dropout 0.1 Fully Connected_1 (Relu) 32<br>Fully Connected_2 (Softmax) 3<br>optimizer: 'Adam', loss: 'categorical_crossentropy', batch_size=8, epochs=150, lr=0.001
**Inception-residual**	Conv1(Relu)8@7×1<br>Conv2_1(Relu)8@1×1 Conv2_2(Relu)8@1×1 Max-pooling 3×1<br>Conv3_1(Relu)5@1×1 Conv3_2(Relu)5@1×1<br>Conv3_3(Relu)5@3×1 Conv3_4(Relu)5@5×1<br>Conv4(Relu)5@1×1<br>Add(Conv4, Conv3_1)<br>Concatenate(Add ,Conv3_2, Conv3_3, Conv3_4)<br>Flatten 3235 Dropout 0.1 Fully Connected_1 (Relu) 32<br>Fully Connected_2 (Softmax) 3<br>optimizer: 'Adam', loss: 'categorical_crossentropy', batch_size=8, epochs=150, lr=0.001

**Inception-attention**	Conv1(Relu)8@7×1<br>Conv2_1(Relu)8@1×1 Conv2_2(Relu)8@1×1 Max-pooling 3×1<br>Conv3_1(Relu)5@1×1 Conv3_2(Relu)5@1×1<br>Conv3_3(Relu)5@3×1 Conv3_4(Relu)5@5×1<br>Concatenate(Add ,Conv3_2, Conv3_3, Conv3_4)<br>Globalaveragepooling Fully Connected_1 (Relu) 2<br>Fully Connected_2 (sigmoid) 5 Reshape (1*1*5) Multiply (Concatenate, Reshape)<br>Flatten 3235 Dropout 0.1 Fully Connected_3 (Relu) 32<br>Fully Connected_4 (Softmax) 3<br>optimizer: 'Adam', loss: 'categorical_crossentropy', batch_size=8, epochs=150, lr=0.001
**Inception-residual-attention**	Conv1(Relu)8@7×1<br>Conv2_1(Relu)8@1×1 Conv2_2(Relu)8@1×1 Max-pooling 3×1<br>Conv3_1(Relu)5@1×1 Conv3_2(Relu)5@1×1<br>Conv3_3(Relu)5@3×1 Conv3_4(Relu)5@5×1<br>Conv4(Relu)5@1×1<br>Add(Conv4, Conv3_1)<br>Concatenate(Add ,Conv3_2, Conv3_3, Conv3_4)<br>Globalaveragepooling Fully Connected_1 (Relu) 2<br>Fully Connected_2 (sigmoid) 5 Reshape (1*1*5) Multiply (Concatenate, Reshape)<br>Flatten 3235 Dropout 0.1 Fully Connected_3 (Relu) 32<br>Fully Connected_4 (Softmax) 3<br>optimizer: 'Adam', loss: 'categorical_crossentropy', batch_size=8, epochs=150, lr=0.001

optimizer: 'Adam', loss: 'categorical_crossentropy', batch_size=8, epochs=150, lr=0.001

batch_size — the number of training samples sent into the network for each training.

epochs — total number of training sessions for all samples.

lr — learning rate
